# Oxygen reduction via grain boundary transport in thin film platinum electrodes on yttria stabilized zirconia

**DOI:** 10.1016/j.ssi.2014.11.006

**Published:** 2015-05

**Authors:** T.M. Huber, A.K. Opitz, J. Fleig

**Affiliations:** Vienna University of Technology, Institute of Chemical Technologies and Analytics, Research Division Electrochemistry, Getreidemarkt 9/164-EC, 1060 Wien, Austria

**Keywords:** Oxygen reduction kinetics, Bulk path, Platinum microelectrodes, Polarization resistance, Grain boundaries

## Abstract

Model-type sputter deposited platinum microelectrodes with different grain sizes were investigated on single crystalline yttria stabilized zirconia (YSZ) by means of impedance spectroscopy. Measurements on single platinum microelectrodes could be continuously performed for > 100 h and from 250 to 800 °C without losing contact. From the temperature dependence, two parallel reaction pathways for oxygen reduction could be identified. Above 450 °C, a surface path with a rate determining step located at the three phase boundary is predominant. Its polarization resistance is independent of the Pt grain size and exhibits an activation energy of ca. 1.8 eV. In the low temperature regime (< 450 °C) a bulk path through Pt was verified, with an electrode polarization resistance depending on the Pt grain size. This resistance is only slightly thermally activated and the rate limiting step is most probably oxygen diffusion along Pt grain boundaries.

## Highlights

• Reaction pathways of O_2_ reduction on Pt/YSZ were investigated by means of impedance spectroscopy.
• Two reaction pathways could be separated by varying the temperature.
• One of these paths was identified to be oxygen transport along Pt grain boundaries.

## 1 Introduction

The system platinum/yttria stabilized zirconia (YSZ) has a long history in both industrial applications and fundamental research. For example, Pt/YSZ is used in oxygen gas sensors applied for exhaust gas analysis in combustion engines [1] and in medical devices [2]. An in-depth understanding of oxygen reduction on Pt/YSZ is crucial for the development of sensors with optimized performance and high reliability [3], [4]. Pt on YSZ is also an important model system for polarization studies in solid oxide fuel cells or solid oxide electrolysis cells (SOFCs or SOECs). Moreover, Pt electrodes are often employed in μ-SOFC [5], [6], [7], [8], [9] based on thin electrolytes and responsible for a significant part of the total polarization resistance of such cells [10].

Pt thin film electrodes are particularly attractive for studies aiming at a mechanistic understanding of the oxygen reduction reaction [11], [12], [13], [14], [15], [16], [17], [18], [19], [20]. Among others, oxygen reduction on Pt thin film microelectrodes has been shown to proceed via two parallel reaction pathways [13]. In a high temperature regime (> 450 °C), a “surface path” was observed, with a rate determining step depending on the three phase boundary (3PB) length. In the lower temperature regime (< 450 °C), a “bulk path” was identified and oxygen transport along platinum grain boundaries (GBs) was assumed to be rate limiting. Indication of oxygen transport along GBs can also be found in Refs. [13], [21], [22], [23]. An unambiguous proof of its importance in oxygen reduction on Pt/YSZ at low temperatures, however, is still missing.

The goal of this work is to analyze the rate limiting step of oxygen reduction on Pt electrodes via the bulk path, i.e. to further clarify the role of oxygen GB diffusion in platinum thin film electrodes. Therefore, electrochemical impedance spectroscopy was performed between 250 and 800 °C on sputter deposited, geometrically well-defined platinum microelectrodes with different grain sizes.

## 2 Experimental

### 2.1 Sample preperation

**Fig. 1:**
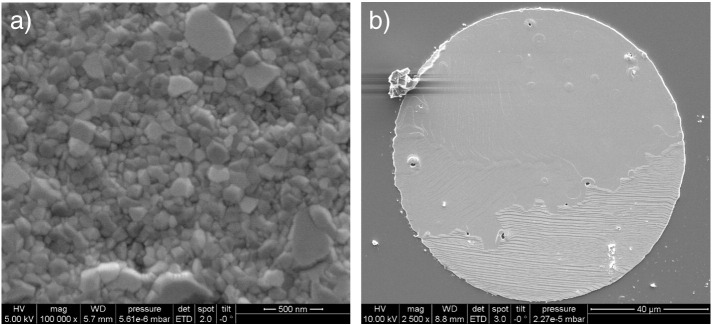
SEM images of platinum microelectrodes a) non-annealed with small grains and b) annealed with large grains.

Platinum thin films were prepared by magnetron sputter deposition (MED 020 Coating System, BAL,TEC, Germany) of Pt (99.95% pure, ÖGUSSA, Austria) on polished YSZ (1 0 0) single crystals (9.5 mol% Y_2_O_3_, CrysTec GmbH, Germany), cf. Ref. [13]. Deposition took place in argon atmosphere at a pressure of 2 × 10^− 2^ mbar without additional heating of the substrate. The film thickness of 350 nm was controlled by means of a quartz micro-balance. Micro-structuring of Pt films was performed by lift-off photolithography (ma-N 1420 negative photo resist and ma-D 533S developer for photo resist, both: micro resist technology, Germany) using a quartz photo mask (Rose, Germany) leading to circular Pt electrodes of different diameters. The size, shape and microstructure of these Pt microelectrodes were investigated by scanning electron microscopy (SEM, FEI Quanta 200 FEG, Netherlands). As counter electrode, Pt paste (Gwent Electronic Materials, UK) was applied onto the back side of the YSZ single crystals. The samples were subsequently annealed in ambient air at 750 °C for 2 h. To obtain electrodes with large grain size, samples were annealed at 800 °C for several weeks. Thereby Pt grains in the thin films grew from initially sub-μm sizes to several 10 μm as shown in Fig. 1. The Ø = 100 μm Pt microelectrode shown in Fig. 1b, for example, has only 3 large grains. From the absolute number of grains visible at the surface of such microelectrodes, a grain size of several 10 μm was estimated. For non-annealed Pt microelectrodes an average grain size of 100–300 nm was estimated from Fig. 1a. Thus, the GB density (total length of grain boundaries visible at the electrode surface) of the small grained Pt microelectrodes is more than 100 times larger than that of electrodes with large grains.

### 2.2 Measurement set-ups

**Fig. 2:**
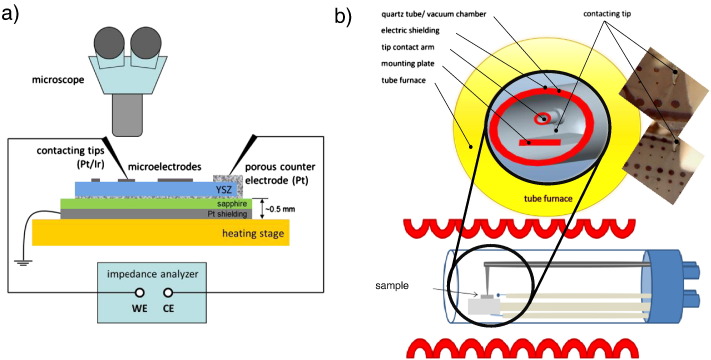
a) Conventional asymmetrically heated micro-contact set-up and b) cross section of the symmetrically heated measurement set-up with photographs of contacted microelectrodes.

Two different micro-contact set-ups were used in the experiments. The asymmetrically heated measurement set-up (Fig. 2a) allows to change the contacted electrode within seconds and thereby to gain statistical information over a large number of different microelectrodes on one and the same sample in a relatively short time. It also enables monitoring of optical changes during the measurement in real time. However, the asymmetrical heating from the bottom side and local cooling (e.g. by convection, radiation, and the contacting tip acting as a heat sink) is known to cause temperature gradients within the sample [11]. Such temperature gradients are responsible for thermo-voltages, which can lead to measurement artifacts in electrochemical experiments [24]. Moreover, in this set-up temperature cycles can hardly be performed on single microelectrodes but require subsequent contacting and de-contacting of different microelectrodes.

The second measurement set-up includes a novel, symmetrically heated sample holder, designed to reduce the influence of temperature gradients; it is sketched in Fig. 2b. It provides a precisely adjustable and measureable sample temperature with minimal temperature gradients in the region of the sample, and it enables long-time measurements on one and the same microelectrode from room temperature up to 1000 °C without losing contact. Since all contacting parts of the symmetrically heated set-up are inside a tube furnace during the measurement, the contacting procedure has to be done prior to the measurement i.e. outside the hot zone. Establishing an electrical contact between tip and microelectrode is performed by a micromanipulator and is monitored by a USB-microscope (Fig. 2b, small photographs). After electrode contacting, the sample is transferred into the hot zone of the tube furnace and fast changes of the contacted electrode are therefore not possible. Detailed information on the two micro-contact set-ups and a discussion on the role of temperature gradients are published elsewhere [24].

### 2.3 Impedance spectroscopy

Electrochemical characterization of the platinum microelectrodes was done by means of two-point impedance measurements using an Alpha-A High Resolution Dielectric Analyzer (Novocontrol, Germany). In both set-ups, electrochemically etched Pt/Ir tips were used to electrically contact the platinum microelectrodes. In the symmetrically heated set-up, the porous platinum counter electrode on the back side of the YSZ substrate was contacted via a Pt sheet beneath the sample (Fig. 2b). In the asymmetrically heated set-up, the counter electrode contact was established on the top side by a second Pt/Ir tip (Fig. 2a). Impedance spectra were recorded at temperatures between 250 and 800 °C in the frequency range of 10^6^ Hz to 10^− 3^ Hz (10^− 2^ Hz in the asymmetrically heated set-up) with a resolution of 5 points per frequency decade.

## 3 Results and discussion

Impedance measurements on annealed electrodes with large grains were performed in the symmetrically heated set-up on several Ø = 200 μm microelectrodes, each contacted once and measured from 250 °C to 800 °C and back to 250 °C with total measurement times much larger than 100 h. The results were then compared with measurements on small grained Pt electrodes, which were conducted in the asymmetrically heated set-up (Fig. 2a) and already reported in an earlier study [13]. Additional cross-check experiments were also performed on large grained electrodes in the asymmetrically heated set-up to exclude systematic errors caused by the use of different set-ups.

**Fig. 3:**
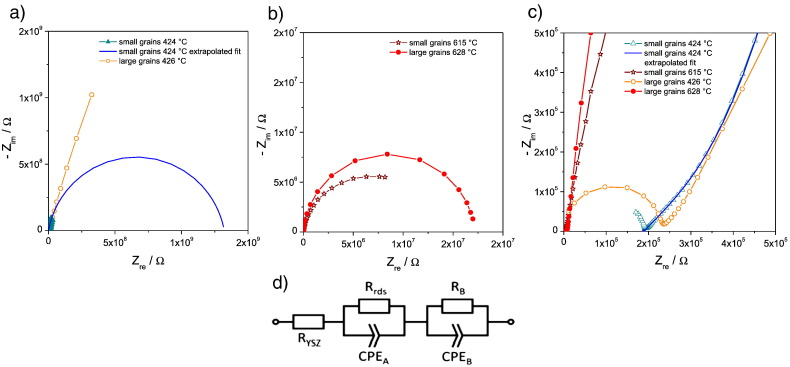
Nyquist plots of two Ø = 200 μm platinum microelectrodes with small and large grains: a) spectra measured at 424 °C and 426 °C, respectively (the blue solid semicircle indicates the extrapolated fit of the measured data (filled triangles). b) Spectra measured at 615 °C and 628 °C, respectively. c) Magnification of the high frequency parts of a) and b) indicating very similar R_YSZ_ for 424 °C/426 °C. d) Equivalent circuit used for the complex nonlinear least square fits of the electrode impedance (without considering the capacitance of the high frequency YSZ bulk arc) and extrapolating to very low frequencies.

Impedance spectra for electrodes with small and large grains are shown in Fig. 3 and both consist of a large semicircle in the low frequency range, a small shoulder in the medium frequency range (Fig. 3c) and a high frequency intercept. For low temperatures, the high frequency intercept, which reflects the spreading resistance of ion conduction in YSZ [11], [25], develops into a more or less complete arc. Its capacitance and thus also its relaxation time is determined by the stray capacitance of the set-up, which exceeds the true bulk capacitance of the microelectrode.

Parameterization of the spectra was carried out by the complex nonlinear least square (CNLS) fit software Z-View2 (Scribner, USA) using the equivalent circuit shown in Fig. 3d. Therein R_ysz_ represents the spreading resistance of ion conduction in YSZ and the two serial R-CPE elements are used to fit the electrode impedance. To account for non-ideal capacitive behavior, constant phase elements (CPE_A_ and CPE_B_) instead of ideal capacitors were employed. The partly or fully developed high frequency (YSZ bulk) arc was not taken into account in the data analysis and the corresponding capacitance is therefore not included in the equivalent circuit. In all cases, R_B_ is several orders of magnitude smaller than the total electrode polarization resistance (R_tot_) and thus negligible compared to the main electrode resistance R_rds_. R_B_ and CPE_B_ were only added to obtain high fit quality and thus being able to extrapolate the spectrum to low frequencies. Hence, the equivalent circuit does not imply a simple mechanistic interpretation of the impedance data. Rather, it is a tool for reliably extrapolating total electrode polarization resistances.

**Fig. 4:**
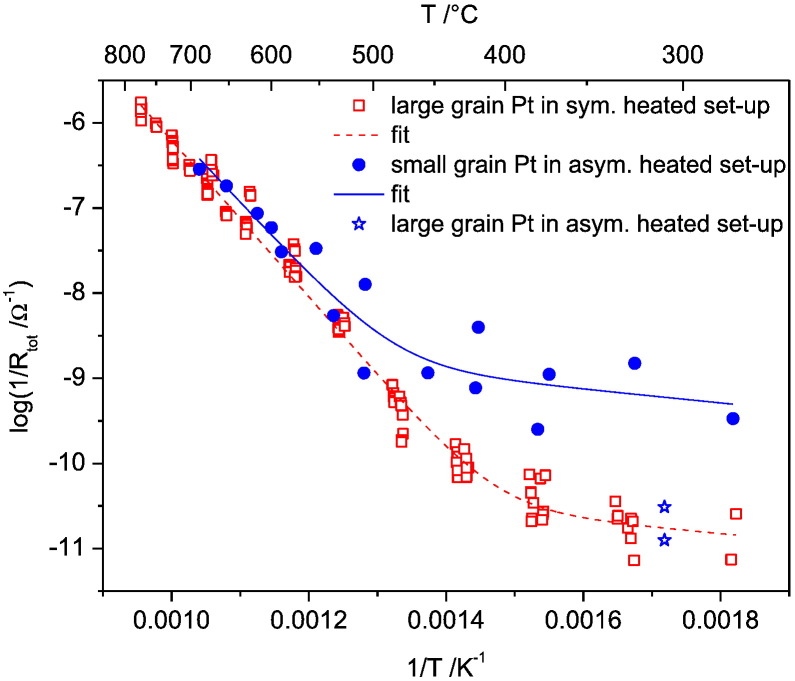
Arrhenius plots of the inverse electrode polarization resistances measured by impedance spectroscopy on Ø = 200 μm platinum microelectrodes. It shows results for electrodes with grain sizes of several 10 μm measured in the symmetrically heated (open squares) and in the asymmetrically heated set-up (stars), and for electrodes with grain sizes of 100–300 nm (filled circles) measured in the symmetrically heated set-up.

At first glance, it might be surprising that reliable electrode polarization resistances can be deduced from the small parts of arcs experimentally accessible at low temperatures in the frequency range under investigation (see Fig. 3a). However, as discussed in detail in Ref. [13] and proven by tests at higher temperatures (with more complete arcs), this extrapolation works rather well due to the well-behaved nature of the interfacial capacitance of the system [13]. The total electrode polarization resistance (R_tot_ = R_rds_ + R_B_ ≈ R_rds_) is plotted in the Arrhenius diagram in Fig. 4, with filled circles and open squares representing the results on electrodes with small and large grains, respectively. Each point of the small grained Pt electrodes is an averaged resistance value obtained on three to five Ø = 200 μm microelectrodes measured three times each; data points for different temperatures include different sets of microelectrodes. The values given for large grained microelectrodes, on the other hand, represent results from entire temperature cycles on several microelectrodes; performing entire cycles on a single microelectrode is possible in the symmetrically heated set-up (see Experimental). These differences in measurement mode and data presentation may also be the reason for the difference in data scattering, which seems more pronounced for small grained electrodes.

In the Arrhenius plot from Fig. 4 both types of Pt microelectrodes—small and large grained—show a qualitatively very similar behavior: a steeper slope (i.e. higher activation energy) at higher temperatures and a much smaller slope at lower temperatures. Moreover, in the high temperature regime also the absolute polarization resistances of both electrode types are similar whereas at low temperatures the total polarization resistances differ by almost two orders of magnitude. Cross-check experiments of large grained electrodes measured in the asymmetrically heated set-up showed that this is not an artifact of the different set-ups (see stars at ca. 320 °C in Fig. 4). To quantitatively analyze both data sets in the Arrhenius plot, a fitting was done by using two parallel admittances Y_1_ and Y_2_ with Y = 1/R. Please note that these two elements (Y_1_ and Y_2_) do not reflect the two resistors R_rds_ and R_B_ from the equivalent circuit but indicate two parallel reaction paths. A detailed argumentation that only two parallel paths can lead to such an Arrhenius plot is given in Ref. [13]. Assuming an exponential temperature dependence of both admittances, the fit function

(1) $$\log\left(Y\right)=\log\left(\frac{1}{R_{\mathrm{tot}}}\right)=\log\left(Y_{1}^{0}\cdot e^{\frac{-Ea_{1}}{k_{B}T}}+Y_{2}^{0}\cdot e^{\frac{-Ea_{2}}{k_{B}T}}\right)$$

results. Therein, the pre-exponential factors Y_1_^0^ and Y_2_^0^ as well as the activation energies Ea_1_ and Ea_2_ are fitting parameters; k_B_ and T denote Boltzmann's constant and temperature, respectively. The fit curves are given in Fig. 4 and the results obtained for the fit parameters are summarized in Table 1.

The fit curves in Fig. 4 and the results in Table 1 confirm that the activation energies for both types of electrodes are almost identical at high temperature. In this temperature region also the pre-exponential factors are in good agreement. At lower temperatures, the slopes and thus the activation energies of electrodes with small and large grains are again virtually identical. The absolute value of ca. 0.15 eV should not be over-interpreted, due to data scattering, but certainly indicates a polarization resistance with only slight thermal activation in both cases. The pre-exponential factors, however, differ by almost two orders of magnitude, suggesting the same rate limiting step for both electrodes but existence of a geometrical scaling factor.

In analogy to Ref. [13], the changing activation energy between low and high temperature regimes can be interpreted in terms of a change of the reaction path and the rate determining step. It was also shown in Ref. [13] that the polarization resistance at high temperatures scales with the 3PB length, while at low temperatures it scales with the electrode surface area. In accordance with this interpretation, both types of electrodes exhibit a similar polarization resistance in the high temperature regime due to a very similar 3PB length. Interestingly, the different electrodes revealed a strongly different response in the low temperature regime, despite having the same surface area. Since the GB density of the two types of electrodes differs by a factor of more than 100 (cf. Sec. 2.1 and Fig. 1), this indicates a crucial role of GBs in the bulk path. Moreover, in one of our prior studies [15], diffusion limited kinetics was found in current voltage measurements between 280 and 400 °C on small grained platinum microelectrodes. The combination of all these observations suggests oxygen diffusion along platinum GBs as the rate determining step of oxygen reduction below 450 °C. Since GB length and 3PB length are similar for large grained microelectrodes, we can draw a further conclusion from the strongly varying temperature dependence: oxygen reduction via 3PBs (active particularly at high temperatures) and oxygen reduction via GBs (dominating at low temperatures) follow very different electrochemical mechanisms. Moreover, the exchange current via the surface path (i.e. the polarization resistance at higher temperature) is independent of the Pt GB density and a pronounced effect of GB/3PB intersections on the oxygen reduction kinetics can thus be excluded.

## 4 Conclusion

Oxygen reduction pathways of sputter deposited platinum microelectrodes with strongly different grain sizes were investigated by means of impedance spectroscopy. Microelectrodes were partly studied in a novel, symmetrically heated micro-contact set-up to avoid temperature gradients within the measured sample. Long-time measurements between 250 and 800 °C could thus be performed on single microelectrodes. Very different activation energies are found at high and low temperatures, irrespective of the grain size. At low temperatures, a bulk path with oxygen transport through Pt is active and its exchange current scales with the grain boundary density of the investigated microelectrodes. This (together with the knowledge of diffusion limited kinetics from a prior study) suggests oxygen diffusion along grain boundaries as a rate limiting step. In contrast, the polarization resistance at high temperatures represents oxygen reduction via a surface path and is not affected by the grain boundary density.

## Figures and Tables

**Table 1:** Activation energies and pre-exponential factors from measurement data and the fit lines in Fig. 4 obtained from Eq. (1).

	Ø = 200 μm Pt electrodes with small grains, measured in asym. heated set-up	Ø = 200 μm Pt electrodes with large grains, measured in sym. heated set-up
Ea_1_ (high temp)	1.71 ± 0.02 eV	1.825 ± 0.003 eV
Ea_2_ (low temp)	0.16 ± 0.02 eV	0.150 ± 0.005 eV
Y^0^_1_	367.5 ± 102.2 Ω^− 1^	983.5 ± 36.9 Ω^− 1^
Y^0^_2_	(1.5 ± 0.5) × 10^−8^ Ω^−1^	(3.5 ± 0.3) × 10^−10^ Ω^−1^
